# Fully automated Bayesian analysis for quantifying the extent and distribution of pulmonary perfusion changes on CT pulmonary angiography in CTEPH

**DOI:** 10.1007/s00330-025-11678-y

**Published:** 2025-05-28

**Authors:** Vojtech Suchanek, Roman Jakubicek, Jan Hrdlicka, Matej Novak, Lucie Miksova, Pavel Jansa, Andrea Burgetova, Lukas Lambert

**Affiliations:** 1 Department of Imaging Methods, Motol University Hospital and Second Faculty of Medicine, Charles University, Prague, Czech Republic; 2 Department of Biomedical Engineering, Faculty of Electrical Engineering and Communication, Brno University of Technology, Brno, Czech Republic; 3 Department of Radiology, First Faculty of Medicine, Charles University and General University Hospital in Prague, Prague, Czech Republic; 4 Second Department of Medicine, First Faculty of Medicine, Charles University and General University Hospital in Prague, Prague, Czech Republic

**Keywords:** Hypertension (pulmonary), Thromboembolism (pulmonary), Computed tomography angiography, Perfusion imaging, Image interpretation (computer-assisted)

## Abstract

**Objectives:**

This work aimed to develop an automated method for quantifying the distribution and severity of perfusion changes on CT pulmonary angiography (CTPA) in patients with chronic thromboembolic pulmonary hypertension (CTEPH) and to assess their associations with clinical parameters and expert annotations.

**Materials and methods:**

Following automated segmentation of the chest, a machine-learning model assuming three distributions of attenuation in the pulmonary parenchyma (hyperemic, normal, and oligemic) was fitted to the attenuation histogram of CTPA images using Bayesian analysis. The proportion of each component, its spatial heterogeneity (entropy), and center-to-periphery distribution of the attenuation were calculated and correlated with the findings on CTPA semi-quantitatively evaluated by radiologists and with clinical function tests.

**Results:**

CTPA scans from 52 patients (mean age, 65.2 ± 13.0 years; 27 men) diagnosed with CTEPH were analyzed. An inverse correlation was observed between the proportion of normal parenchyma and brain natriuretic propeptide (proBNP, ρ = −0.485, *p* = 0.001), mean pulmonary arterial pressure (ρ = −0.417, *p* = 0.002) and pulmonary vascular resistance (ρ = −0.556, *p* < 0.0001), mosaic attenuation (ρ = −0.527, *p* < 0.0001), perfusion centralization (ρ = −0.489, *p* = < 0.0001), and right ventricular diameter (ρ = −0.451, *p* = 0.001). The entropy of hyperemic parenchyma showed a positive correlation with the pulmonary wedge pressure (ρ = 0.402, *p* = 0.003). The slope of center-to-periphery attenuation distribution correlated with centralization (ρ = −0.477, *p* < 0.0001), and with proBNP (ρ = −0.463, *p* = 0.002).

**Conclusion:**

This study validates an automated system that leverages Bayesian analysis to quantify the severity and distribution of perfusion changes in CTPA. The results show the potential of this method to support clinical evaluations of CTEPH by providing reproducible and objective measures.

**Key Points:**

***Question*** *This study introduces an automated method for quantifying the extent and spatial distribution of pulmonary perfusion abnormalities in CTEPH using variational Bayesian estimation*.

***Findings*** *Quantitative measures describing the extent, heterogeneity, and distribution of perfusion changes demonstrate strong correlations with key clinical hemodynamic indicators*.

***Clinical relevance*** *The automated quantification of perfusion changes aligns closely with radiologists’ evaluations, delivering a standardized, reproducible measure with clinical relevance*.

**Graphical Abstract:**

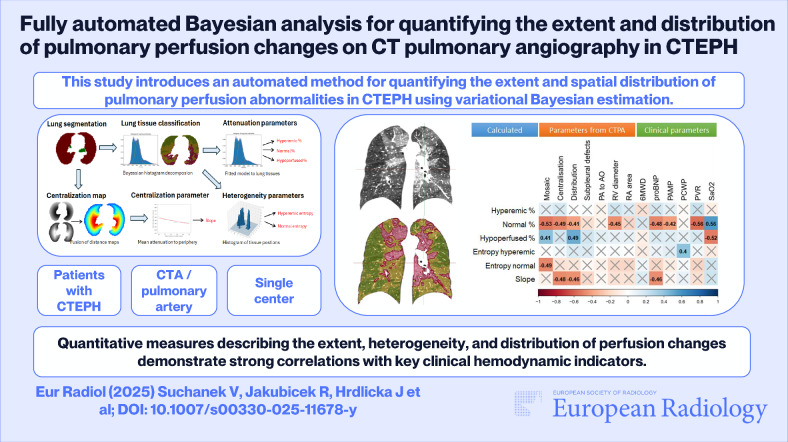

## Introduction

Chronic thromboembolic pulmonary hypertension (CTEPH) is characterized by symptomatic persistent obstruction or stenosis of the pulmonary arteries due to organized thrombi and secondary microvasculopathy, resulting in progressive pulmonary hypertension and right heart failure if left untreated [1]. Computed tomography pulmonary angiography (CTPA) is essential for evaluating the extent and characteristics of CTEPH, depicting structural changes in the pulmonary artery (PA) and changes in pulmonary perfusion. Perfusion defects in the lungs observed on ventilation/perfusion (V/Q) scans are fundamental for diagnosis, although CTPA provides similar diagnostic accuracy and simultaneous assessment of the PA, pulmonary parenchyma, and perfusion [2].

Mosaic perfusion with central hyperemic areas and peripheral oligemia is indicative of remodeling in the periphery of PA (small vessel disease) and implies poor prognosis [3]. The traditional methods used to analyze mosaic perfusion on CTPA are largely subjective and rely on expert interpretation, which is prone to variability [4–6]. To date, there has been no automated method for quantifying the extent of perfusion changes and their distribution on CTPA.

The aim of this study was to develop an automated method for quantifying the extent and distribution of perfusion changes on conventional CTPA in patients with CTEPH and to assess their associations with clinical parameters and expert annotations.

## Materials and methods

This retrospective study was carried out in accordance with the Declaration of Helsinki (seventh revision). The Ethics Committee stated that the study required neither its approval nor informed consent (198/22 S-IV).

### Patient population

In this study, we included consecutive patients who were diagnosed with CTEPH between January 1, 2018, and December 31, 2018, in the Cardiology Department of a tertiary academic hospital with a national CTEPH referral center. The exclusion criteria were incomplete data, malignancy, interstitial lung disease, and the unavailability of thin slices from CTPA. The clinical data (shown in Table 1) were retrieved from the hospital information system.

**Table 1:** Patient characteristics

Variable	Value
*n*	52
Age [years]	65.2 ± 13.0
Gender [male, *n* (%)]	27 (52%)
Height [cm]	170 (IQR: 163–182)
Weight [kg]	82 (IQR: 73–93)
BMI [kg·m^−2^]	28.4 (IQR: 24.5–31.7)
BSA [m^2^]	1.93 (IQR 1.80–2.06)
NYHA class	3 (IQR: 2–3)
6MWD [m]	398 ± 111
proBNP ng/L	1010 (IQR: 158–2954)
Right atrium [cm^2^]	7 (IQR: 4–10)
PAMP [mmHg]	43 (IQR: 35–55)
PCWP [mmHg]	10 (IQR: 7–12)
CO [L·min^−1^]	4.27 (IQR: 3.505–5.218)
CI [L·min^−1^·m^−2^]	2.23 ± 0.41
PVR [wood]	8.01 ± 3.55
HR [min^−1^]	70.8 ± 12.4
SaO_2_ [%]	93.5 (IQR: 90.2–96.1)
SvO_2_ [%]	64.2 ± 9.0
FEV1 [%]	88.3 ± 15.9
FVC [% norm]	95.7 ± 17.3
EF LV [%]	62.3 ± 7.0

Values are presented as mean ± standard deviation or median (IQR) according to their distribution

*IQR* interquartile range, *BSA* body surface area, *NYHA* New York Heart Association, *6MWD* 6 min walking distance, *proBNP* brain natriuretric peptide prohormone, *PAMP* mean pulmonary arterial pressure, *PCWP* pulmonary capillary wedge pressure, *CO* cardiac output, *CI* cardiac index, *PVR* pulmonary vascular resistance, *HR* heart rate, *SaO*_2_ arterial oxygen saturation, *SvO*_2_ mixed venous oxygen saturation, *FEV1* forced expiratory volume in the first second, *FVC* forced vital capacity, *EF LV* ejection fraction of the left ventricle

During short-term hospitalization, V/Q scan, CTPA, echocardiography, exercise capacity testing, pulmonary function tests, laboratory tests, PA catheter angiography, and hemodynamic studies were performed. The final diagnosis of CTEPH was based on the criteria of the European Society of Cardiology [1].

### CTPA technique

CT examinations were performed on a 256-slice CT scanner (iCT Brilliance, Philips) in craniocaudal spiral acquisition (peak tube voltage 100 kV, planned tube current-time product 230 mAs/slice, rotation time 0.5 s, pitch factor 0.993, and collimation of 128 × 0.625 mm) and reconstructed as 0.9-mm slices with 50% overlap using iterative reconstruction (iDOSE^4^). Bolus tracking with a threshold of 150 HU in the PA was used as a triggering method. Contrast agent, 65 mL of Optiray 350 mg I/mL (Guerbet) or Iomeron 350 mg I/mL (Bracco), was administered intravenously at a flow rate of 4.0 mL/s using an automated CT injection system (Medrad® Stellant, Bayer), followed by a 40 mL saline flush at the same flow rate.

### Multiparametric CTPA analysis

CTPA images were evaluated on a diagnostic workstation (Philips IntelliSpace Portal v.10, CT viewer). Two radiologists (J.H., M.N.) with 4- and 9-years’ of cardiovascular imaging experience measured the following parameters: diameters of the PA and ascending aorta, areas of the right and left atria in four-chamber view, and right and left chamber diameters in four-chamber view. The following parameters were evaluated on a four-point Likert scale (4PS): the presence of mosaic perfusion, perfusion centralization, peripheral distribution of perfusion defects, subpleural perfusion deficit, PA tree involvement at the central, (sub)segmental, and peripheral level (0, absent; 1, rather absent; 2, rather present; and 3, present) [7]. The average values and scores were used in the analysis.

### Automated analysis of perfusion changes

**Fig. 1:**
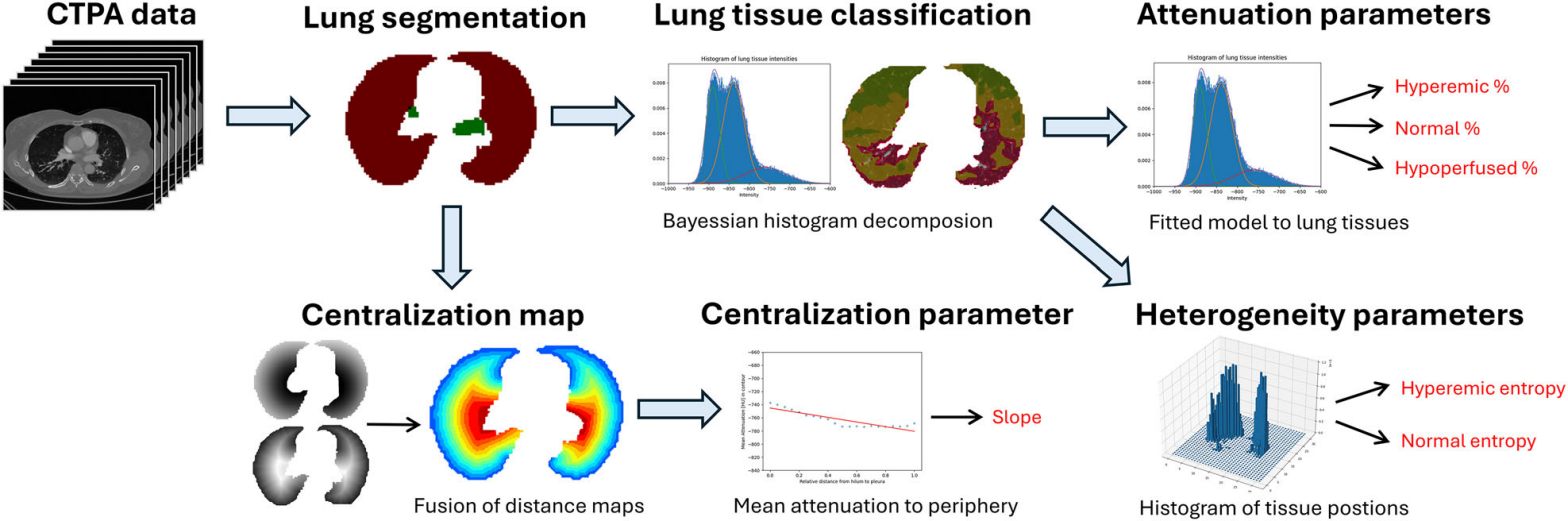
Process diagram of automated image analysis

The trained Total Segmentator network, which is publicly available on GitHub, was used to segment the lungs and pulmonary veins [8]. The segmentation results were visually checked for accuracy. All three main analyses were implemented in Python 3.12, and the software was fully automated. The software was made available on GitHub (https://github.com/JakubicekRoman/lung_CTPA.git). A graphical process diagram is shown in Fig. 1.

### Attenuation analysis

**Fig. 2:**
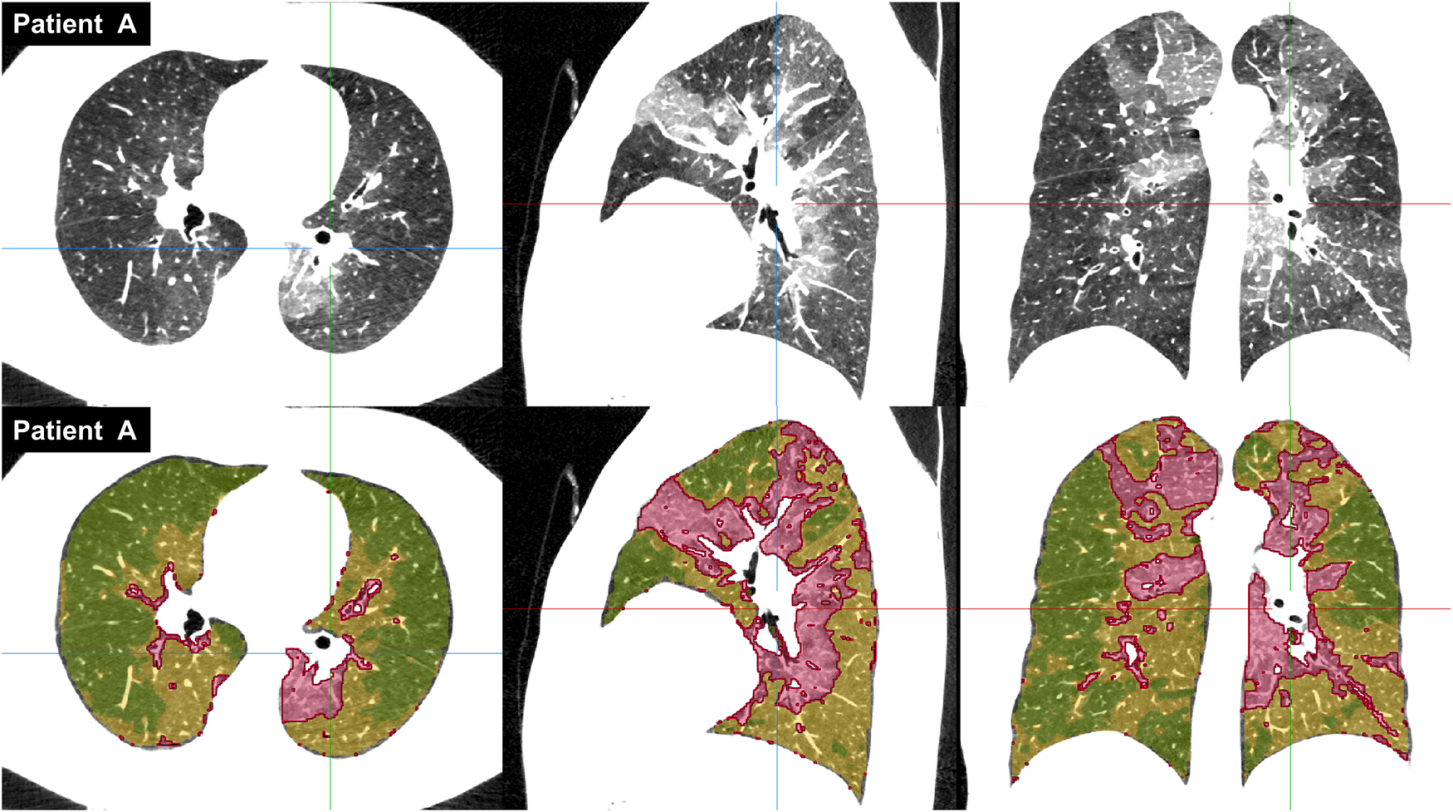
Example of tissue classification based on histogram decomposition in Fig. 4, Patient A. Three tissues (high, medium, and low attenuation) on CTPA in the axial, sagittal, and coronal planes (above) are distinguished by color (red, yellow, and green) (below)

**Fig. 3:**
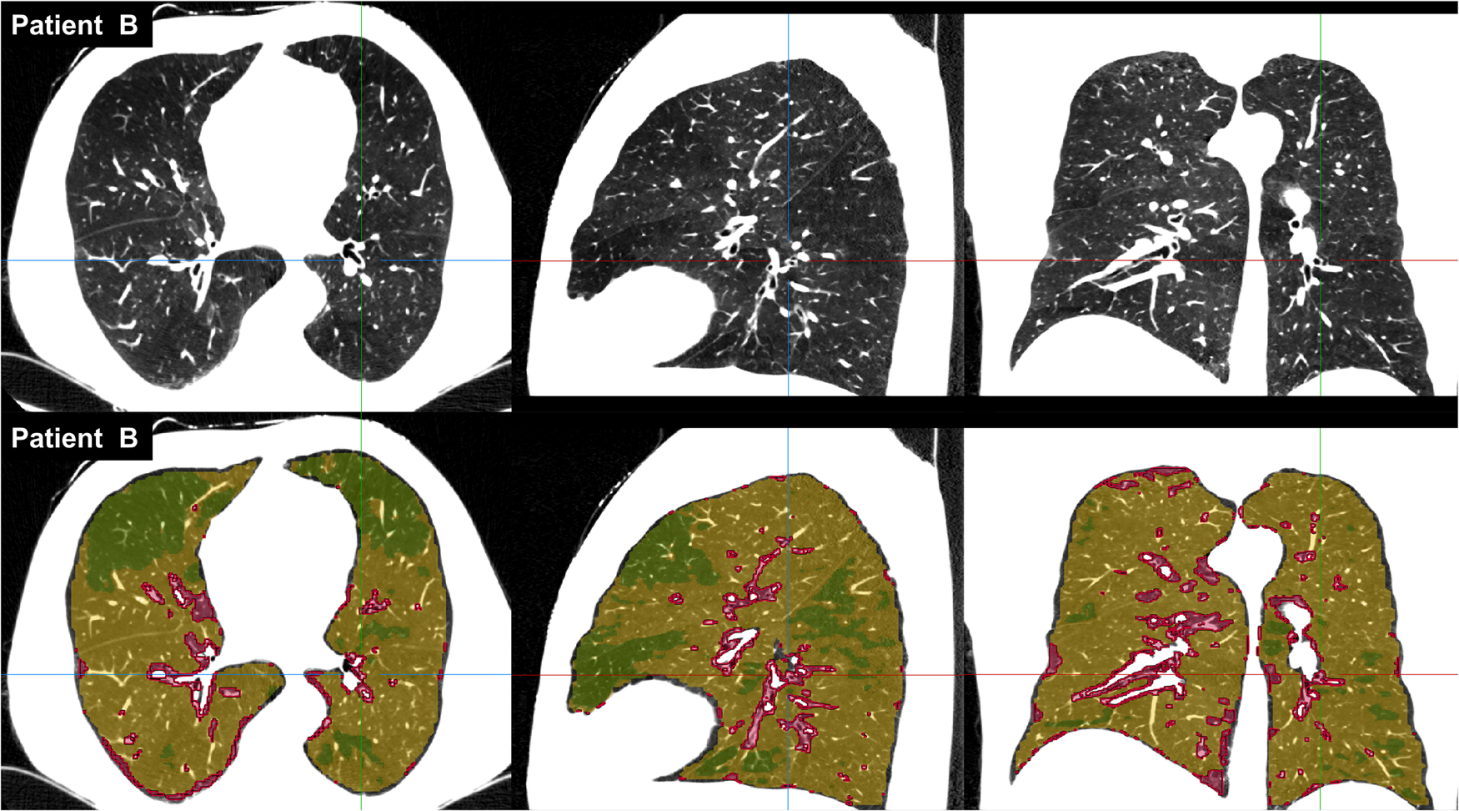
Example of tissue classification based on histogram decomposition in Fig. 4, Patient B with minimal perfusion changes. Three tissues (high, medium, and low attenuation) on CTPA in the axial, sagittal, and coronal planes (above) are distinguished by color (red, yellow, and green) (below)

**Fig. 4:**
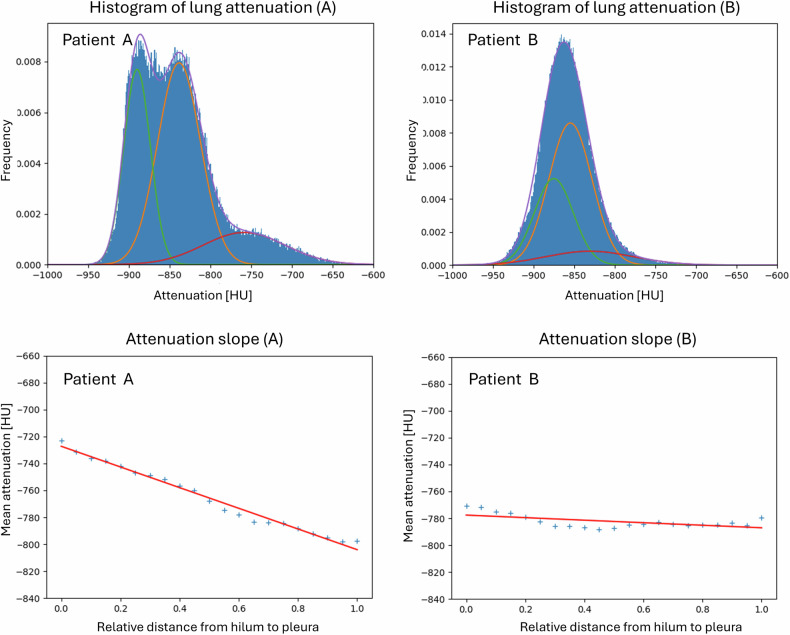
Histogram of lung attenuation with three fitted Gaussian distributions representing high (red), medium (orange), and low (green) attenuation in a patient with severe perfusion changes, central hyperemia and peripheral oligemia (Patient A, left) and in a patient with minimal perfusion changes (Patient B, right) (above). Graph of mean attenuation vs relative distance from the hilum to the pleura (blue crosses) and fitted line (red) in a patient with severe perfusion changes (Patient A, left) and minimal perfusion changes (Patient B, right) (below)

This model assumes three compartments in the lungs according to attenuation (high = hyperemic, medium = normal, and low = oligemic) (Figs. 2–4) [9]. Using variational Bayesian estimation, a Gaussian mixture model representing the sum of three individual Gaussian distributions is fitted to the histogram of voxel intensities of both lungs [10]. This approach minimizes the potential bias caused by contrast enhancement variability. Each distribution is then characterized by its relative proportion in the histogram. The parameterized model, according to the histogram of a specific patient, is then applied to the CT data, providing a mask of classifications of individual voxels.

### Heterogeneity analysis

For the high- and medium-attenuation compartments of the three-compartment model, we calculated the entropy of the 3D histogram of voxel spatial coordinates. The histograms of both compartments were computed for 32 × 32 × 32 bins in each lung separately. Then their entropy values were calculated. The results were averaged between the two lungs. These parameters reflect spatial heterogeneity, i.e., dispersion of areas of the specific compartment.

### Centralization analysis

To analyze the distribution of attenuation between the central and peripheral regions, the slope of the fitted linear curve of mean attenuation vs the relative distance from the hilum to the periphery was automatically calculated (Fig. 4). The relative distance from the hilum to the periphery is computed using two distance maps. The first distance map is derived from the lung contours obtained from the lung segmentation mask, representing the lung periphery. The second distance map is based on the detected hilar point, which is determined as the average coordinates of the entry of the pulmonary veins located outside the lung. By normalizing the ratio of these two distance maps, we obtain the centralization map, which reflects the relative distance from the periphery to the hilum (Fig. 1). To determine the slope of the fitted linear curve, we then calculated a set of 21 average attenuation values in specific regions (contours) from central to peripheral regions controlled by the centralization map. The fitting of the linear curve was performed using a polynomial least mean square fitting algorithm. The resulting slope values were averaged between the two lungs.

### Statistical analysis

Statistical analysis was performed in R and SPSS v. 19 (IBM Corp.) [11]. The values were represented as mean ± standard deviation or median (interquartile range, IQR) according to distribution (D’Agostino normality test). The Grubbs test and data plots were used to detect outliers. Spearman’s rank correlation coefficient (ρ) among the calculated parameters, clinical data, and measurements performed by the radiologists was calculated.

A *p*-value below 0.05 was considered significant. For multiple comparisons, a false discovery rate correction was made (Benjamini–Hochberg). For 13 variables [mosaic attenuation, perfusion centralization, distribution of perfusion defects, subpleural perfusion deficit, PA to ascending aorta diameter ratio, right ventricle diameter, right atrium area, 6-min walking distance, brain natriuretic peptide prohormone (proBNP), mean PA pressure (PAMP), pulmonary capillary wedge pressure (PCWP), pulmonary vascular resistance (PVR), arterial oxygen saturation], the corrected critical *p*-value was 0.0042.

## Results

The CTPAs of 52 patients (mean age, 65.2 ± 13.0 years; 27 men, 25 women) were analyzed (Table 1, Supplementary Tables 1 and 2, and Supplementary Fig. 1).

**Fig. 5:**
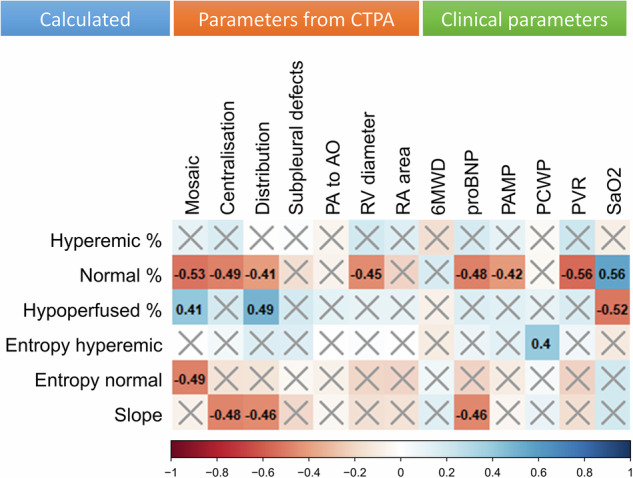
Cross-correlation matrix. The color scale and numbers represent Spearman’s correlation coefficients (−1 to 1). Only coefficients with *p*-values below 0.0042 are shown (false discovery rate correction). PA, pulmonary artery; AO, aorta; RV, right ventricle; RA, right atrium; 6MWD, 6 min walking distance; proBNP, brain natriuretic peptide prohormone; PAMP, mean PA pressure; PCWP, pulmonary capillary wedge pressure; PVR, pulmonary vascular resistance; SaO_2_, arterial oxygen saturation

The attenuation analysis revealed a negative correlation between the proportion of moderately attenuating (normal) parenchyma and proBNP (ρ = −0.485 *p* = 0.001), PAMP (ρ = −0.417, *p* = 0.002), PVR (ρ = −0.556, *p* < 0.0001), mosaic attenuation (ρ = −0.527, *p* < 0.0001), centralization (ρ = −0.489, *p* < 0.0001), and right ventricular (RV) diameter (ρ = −0.451, *p* = 0.001, Fig. 5).

In the heterogeneity analysis, a negative correlation was found between the entropy of the moderately attenuating (normal) parenchyma and mosaic pattern as evaluated by radiologists (ρ = −0.486, *p* < 0.0001). The entropy of the highly attenuating (hyperemic) parenchyma was positively correlated (ρ = 0.402, *p* = 0.003, Fig. 5) with the PCWP.

The slope of attenuation from the hilum to the pleura correlated with centralization (ρ = −0.477, *p* < 0.0001) and the distribution of these changes (ρ = −0.456, *p* = 0.001) rated by radiologists and also with proBNP (ρ = −0.463, *p* = 0.002, Fig. 5).

## Discussion

In this study, we developed an algorithm to automatically quantify three features of perfusion changes in CTPA in patients with CTEPH. We showed that the proportion of moderately attenuating (normal) parenchyma and its spatial distribution inversely correlate with radiologists’ perception of mosaic attenuation, centralization, peripheral oligemia, and also with clinical parameters. This means that the automated calculation of these parameters quantifies what radiologists perceive and reflects the severity of the disease described by clinical and hemodynamic assessment.

Perfusion defects are a hallmark of CTEPH, with 100% sensitivity and 95% specificity [12]. Perfusion changes can be recognized not only on V/Q scintigraphy but also on unenhanced CT and CTPA with and without dual energy acquisition [6, 13, 14]. The dual energy lung blood volume uses material decomposition to quantify the iodine content in the lung parenchyma [14]. The results of iodine maps in patients with CTEPH have shown a strong correlation with mosaic attenuation on standard CTPA, a method that is easily interpretable even by general radiologists [7, 15]. In CTEPH, remodeling of the peripheral vasculature, called small vessel disease, results in centralization of perfusion and hypoperfusion of the periphery, which are associated with a poor prognosis [3, 16].

The quantification of regions of different attenuations from both unenhanced and contrast-enhanced CT has been attempted previously in different scenarios. Romanov et al separated attenuation bins from high-resolution CT (HRCT) images using attenuation thresholds of 0 HU, −250 HU, −500 HU, −600 HU, −700 HU, and −800 HU and evaluated their relative proportions [9]. The frequency distribution of the attenuation bins was described by the mean, standard deviation, and skewness and was used to separate normal parenchyma and atypical pneumonia. Histogram analysis has been used in other applications, such as pulmonary emphysema, pulmonary fibrosis, and even CTEPH [17–19]. For the quantification of emphysema and fibrosis in HRCT, textural analysis has shown superiority to histogram-based methods even in longitudinal trials [20].

These approaches assume a priori knowledge about texture and attenuation and, therefore, have limited portability to other areas, including contrast-enhanced studies. In our study, we proposed a machine-learning model that classifies hyperemic, normal, and oligemic parenchyma using Bayesian estimation to fit each of the three individual Gaussian distributions to the lung attenuation histogram [9]. This unsupervised learning model offers greater flexibility and has not yet been applied in this context. In contrast to other unsupervised clustering methods used for density estimation in areas such as the breast or brain, which divide distributions more sharply, the Bayesian approach of the Gaussian mixture model assigns tissue bins based on normal probability distributions, allowing for more accurate predictions of real density distributions [21, 22].

The quantification of perfusion changes by measuring the volume of the hypoperfused, normally perfused, and hyperperfused parenchyma reflects the ratings of various aspects of mosaic perfusion, including its severity and distribution by radiologists. The entropy of areas of the normally perfused parenchyma correlates with what radiologists classically describe as mosaic attenuation. The gradient of attenuation from the center to the periphery correlates with the description of the centralization of circulation and its distribution between the central and peripheral parts of the lungs. Furthermore, automatically calculated quantitative parameters are correlated with clinical characteristics that indicate the severity of the disease and predict outcomes [23].

A deep learning approach to tissue segmentation and characterization addresses the limitations of fixed thresholding-based methods by employing unknown and more complex features and relationships that can more accurately characterize diseased tissue and predict patient outcomes [24]. However, the extracted features are highly abstract and nonlinear, making their interpretation challenging. In contrast, the extracted image and shape features in our approach are physically derived and therefore comprehensible. Moreover, deep-learning approaches require large cohorts and data annotations, which are difficult to obtain in CTEPH.

### Study limitations

This study has several limitations. First, this is a monocentric study performed on a single CT scanner with a single population. The lack of an external validation cohort poses a challenge to generalizability. Second, a strictly three-parameter model composed of three Gaussian distributions may not be accurate in healthy individuals, where the proportion of hyper- or hypoperfused pulmonary parenchyma is minimal. This would also affect the heterogeneity analysis, which relies on the separation of the three components. Additionally, high attenuating artifacts may be misclassified as hyperperfused regions. Third, the centralization analysis is dependent on the Total Segmentator results, which provided correct segmentation in all patients.

## Conclusions

An automated system for the quantification of perfusion changes and their distribution on CTPA has been validated with radiologists’ annotations and correlations with clinical characteristics of CTEPH. This system relies on a three-compartment model and Gaussian fitting of the distribution of the lung attenuation histogram, which adopts flexibility in estimating tissue boundaries.

## Supplementary information

ELECTRONIC SUPPLEMENTARY MATERIAL

## Abbreviations

CTEPH: Chronic thromboembolic pulmonary hypertension
CTPA: Computed tomography pulmonary angiography
PA: Pulmonary artery
