# Supplementary material for: Fully automated Bayesian analysis for quantifying the extent and distribution of pulmonary perfusion changes on CT pulmonary angiography in CTEPH
Source: Eur Radiol. 2025 May 28;35(11):6996–7003. doi: 10.1007/s00330-025-11678-y (PMC12559064; doi:10.1007/s00330-025-11678-y)

**Fully automated Bayesian analysis for quantifying the extent and distribution of pulmonary perfusion changes on CT pulmonary angiography in CTEPH**

ELECTRONIC SUPPLEMENTARY MATERIAL

**Suppl. Table 1.** Patient comorbidities.

| **Variable** | **Number** | **Percent** |
| --- | --- | --- |
| **Pacemaker** | 2 | 4 % |
| **Chronic obstructive lung disease** | 13 | 25 % |
| **Asthma** | 0 | 0 % |
| **Atrial fibrillation** | 11 | 21 % |
| **Atrial flutter** | 4 | 8 % |
| **Ischemic heart disase** | 9 | 17 % |
| **Stroke** | 4 | 8 % |
| **Hypertension** | 28 | 54 % |
| **Diabetes mellitus** | 9 | 17 % |
| **Peripheral arterial disease** | 5 | 10 % |
| **Ex-smoker** | 23 | 44 % |
| **Smoker** | 3 | 6 % |

**Suppl. Table 2.** Parameters scored or measured on CTPA.

| **Variable** | **Value** |
| --- | --- |
| Mosaic perfusion (0-3)^1^ | 3.0 (IQR 2.0-3.0) |
| Perfusion centralization (0-3) ^1^ | 2.0 (IQR 1.0-2.8) |
| Peripheral distribution of perfusion defects (0-3)^1^ | 1.8±0.9 |
| Subpleural perfusion deficit (0-3)^1^ | 1.6±0.9 |
| AA diameter (mm) | 33.5 (IQR 30.0-36.0) |
| AA diameter BSA | 17.2 (IQR 14.9-18.6) |
| PA diameter (mm) | 32.0 (IQR 30.0-35.0) |
| PA diameter BSA | 17.3±2.8 |
| PA to AO diameter ratio | 1.0±0.2 |
| RV diameter (mm) | 47.4±9.9 |
| RV diameter BSA | 24.8±5.6 |
| RA area (cm^2^) | 26.5 (IQR 21.0-34.0) |
| RA area BSA | 14.4 (IQR 11.0-19.0) |

Values presented as mean±standard deviation or median (IQR) according to their distribution.

IQR, interquartile range; BSA, body surface area; AA, ascending aorta; PA, pulmonary artery; RV, right ventricle; RA, right atrium.

^1^0=absent, 1= rather absent, 2=rather present, 3=present

**Suppl. Table 3.** Cross-correlation matrix showing Spearman’s (ρ) correlation coefficients (-1 to 1) and p values (values below 0.0042 are considered significant).

| **Calculated parameters** | **Mosaic perfusion** | | **Perfusion centralisation** | | **Perfusion distribution** | | **Subpleural defects** | | **PA to AO** | | **RV diameter** | | **RA area** | |  | **6MWD** | | **proBNP** | | **PAMP** | | **PCWP** | | **PVR** | | **SvO2** | |
| --- | --- | --- | --- | --- | --- | --- | --- | --- | --- | --- | --- | --- | --- | --- | --- | --- | --- | --- | --- | --- | --- | --- | --- | --- | --- | --- | --- |
|  | ρ | p | ρ | p | ρ | p | ρ | p | ρ | p | ρ | p | ρ | p |  | ρ | p | ρ | p | ρ | p | ρ | p | ρ | p | ρ | p |
| **Hyperemic %** | 0.216 | 0.125 | 0.304 | 0.029 | 0.03 | 0.833 | 0.046 | 0.747 | -0.12 | 0.399 | 0.353 | 0.01 | 0.274 | 0.05 |  | -0.295 | 0.061 | 0.328 | 0.03 | 0.214 | 0.132 | -0.069 | 0.633 | 0.392 | 0.006 | -0.19 | 0.191 |
| **Normal %** | -0.527 | <0.0001 | -0.489 | <0.0001 | -0.405 | 0.003 | -0.291 | 0.036 | -0.135 | 0.339 | -0.451 | 0.001 | -0.375 | 0.006 |  | 0.31 | 0.048 | -0.485 | 0.001 | -0.417 | 0.002 | -0.076 | 0.597 | -0.556 | <0.0001 | 0.564 | <0.0001 |
| **Hypoperfused %** | 0.414 | 0.002 | 0.343 | 0.013 | 0.49 | <0.0001 | 0.348 | 0.011 | 0.235 | 0.094 | 0.206 | 0.143 | 0.206 | 0.142 |  | -0.156 | 0.33 | 0.283 | 0.063 | 0.253 | 0.073 | 0.209 | 0.141 | 0.303 | 0.036 | -0.52 | <0.0001 |
| **Entropy hyperemic** | 0.004 | 0.975 | 0.103 | 0.469 | 0.288 | 0.038 | 0.27  Parameters obtained from CTPA | 0.053 | 0.017 | 0.905 | 0.049 | 0.73 | -0.008 | 0.957 |  | -0.206 | 0.197 | 0.153 | 0.32 | 0.249 | 0.078 | 0.402 | 0.003 | 0.129 | 0.383 | -0.212 | 0.143 |
| **Entropy normal** | -0.486 | <0.0001 | -0.278 | 0.046 | -0.259 | 0.064 | -0.089 | 0.529 | -0.144 | 0.31 | -0.354 | 0.01 | -0.361 | 0.009 |  | 0.134 | 0.403 | -0.38 | 0.011 | -0.203 | 0.153 | 0.082 | 0.567 | -0.385 | 0.007 | 0.353 | 0.013 |
| **Slope** | 0.147 | 0.3 | 0.477 | <0.0001 | 0.456 | 0.001 | 0.34 | 0.014 | 0.095 | 0.501 | 0.287 | 0.039 | 0.27 | 0.053 |  | -0.268 | 0.091 | 0.463 | 0.002 | 0.11 | 0.443 | -0.188 | 0.187 | 0.296 | 0.041 | -0.363 | 0.01 |
|  | **Parameters obtained from CTPA** | | | | | | | | | | | | | |  | **Clinical parameters** | | | | | | | | | | | |

PA, pulmonary artery; AO, aorta; RV, right ventricle; RA, right atrium; 6MWD, 6 minute walking distance; proBNP, brain natriuretric peptide prohormone; PAMP, mean PA pressure; PCWP, pulmonary capillary wedge pressure; PVR, pulmonary vascular resistance; SvO_2_, mixed venous oxygen saturation.

**Suppl. Figure 1.** Pulmonary artery tree involvement at central, segmental and subsegmental, and peripheral levels.


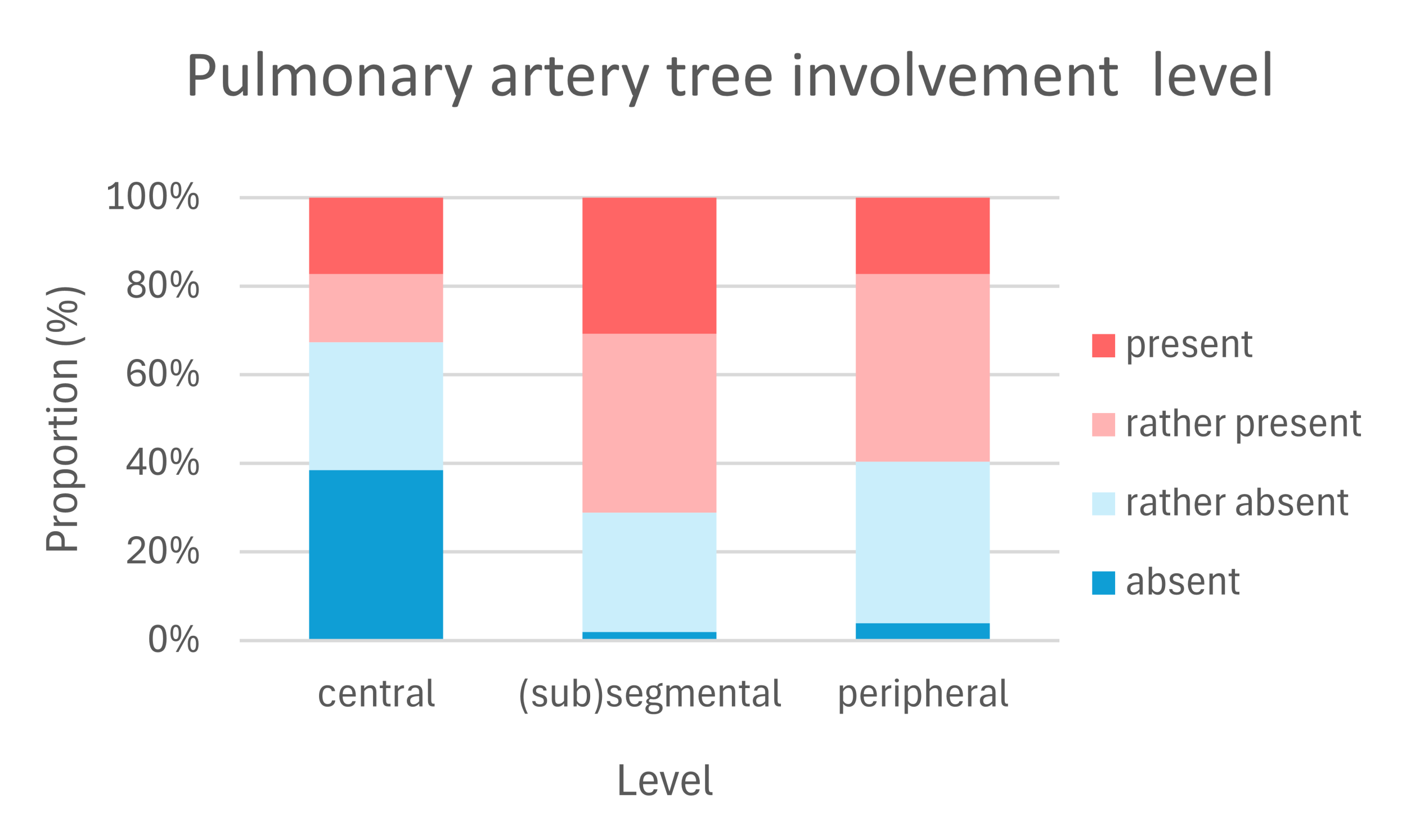

Supplement: Supplementary file 1 — ELECTRONIC SUPPLEMENTARY MATERIAL [file 330_2025_11678_MOESM1_ESM.docx]
